# Bone quality effect on short implants in the edentulous mandible: a finite element study

**DOI:** 10.1186/s12903-022-02164-8

**Published:** 2022-04-26

**Authors:** Chaowei Liu, Yifeng Xing, Yan Li, Yanjun Lin, Jianghan Xu, Dong Wu

**Affiliations:** 1 Provincial Engineering Research Center of Oral Biomaterial, Fujian Medical University, Fuzhou, 350001 China; 2 School and Hospital of Stomatology, Fujian Medical University, Fuzhou, 350001 China; 3 Research Center of Dental and Craniofacial Implants, Fujian Medical University, Fuzhou, 350001 China

**Keywords:** Short implants, Finite element analysis, Bone quality, Edentulous jaw

## Abstract

**Introduction:**

The aim of this study was to verify whether the use of short implants could optimize stress distribution of bone surrounding implants in atrophic mandibles with different bone qualities.

**Methods:**

A three-dimensional model of the atrophic mandible with three levels of bone quality was made using computer software. Short implants (6 mm) and standard implants (10 mm) were used in four designs: Design 1 "All-On four", Design 2 "All-On-four" with two short implants, Design 3 four vertical implants with two short implants, and Design 4 six short implants. The distal short implants were placed at the first molar position. All twelve models were imported into finite element analysis software, and 110 N oblique force was loaded on the left second premolar. Maximum principal stress values of peri-implant bone and the volumes of bone with over 3000 microstrians (overload)were analyzed.

**Result:**

Stress values and volumes of overload bone increased in all four groups with the decline of bone quality. The highest stress values were found in the cortical bone surrounding the Design 1 inclined implant in two lower bone quality mandibles, and the lowest in Design 3. However, Design 1 had less overload bone tissue than all three designs with short implants.

**Conclusion:**

Short implants placed posteriorly helped decrease stress values in peri-implant bone, while bone surrounding short implants had a high resorption risk in low bone quality mandible.

## Introduction

Dental implants have become the best choice for prosthodontic restoration of edentulous jaws, for significantly recovering the masticatory ability with long-term stable results. However, alveolar bone resorption brings more challenges to the implant inserting, such as bone augmentation procedures, increasing the cost and complication risks [1, 2].

Maló’s “All-on-4” concept, in which four implants are placed between mental foramens with the posterior two tilted, avoids bone resorption areas and gains sufficient bone for standard implants, presenting a high success rate [3]. Tilted placed implants eliminate the need for bone grafting, while bone tissue around them suffers higher stress than around the vertical implants [4]. At the same time, the distal cantilever can cause greater deformation in the superstructures and induce mechanical complications [5, 6].Therefore, more alternative schemes still need to be found.

With the improvement of implant surface treatment methods, more implant specifications are available. At present, short implants are usually considered to be less than 6–8 mm [7].They can replace long implants in atrophic alveolar bone, with less procedure, cost and time [8].

Short implants have been used in fully edentulous jaws as a supplement, and the design even can be seen that full-arch prostheses are supported all by short implants [9]. In some biomechanics studies, compared with traditional “all-on-4” concept, the addition of short implants in the posterior area helps decrease the stress in peri-implant bone [10, 11].The use of short implants in edentulous jaw restoration seems to be worth a try.

There are several methods to analyze the stress of dental implants and bone, including in vitro and in vivo strain gauge tests, photoelastic analysis and finite element analysis (FEA) [12–14]. FEA is the most commonly used method because it can simulate different complex situations and provide rich information. In the previous FEA studies about short implants, the cortical bone and cancellous bone were modeled respectively to better simulate the actual situation of the jaw, but the whole jaw was regarded as the same setting, which may mislead the use of short implants in the posterior areas. Actually, there are differences in bone quality between the anterior and posterior jaws [15]. Bone quality has been proved to associate with implant survival rate, and the implant failure rate is higher under low bone quality [16–18]. Some FEA studies show that the stress and strain of the peri-implant bone are negatively correlated with the length and diameter of the implant [19–21]. Therefore, there may be some potential risks in the use of short implants in the posterior jaws with low bone quality.

Given the possible influence of bone quality on short implants and the inadequacy of current literature, this study aimed to verify whether short implants in atrophic mandibles with different bone qualities optimize the stress distribution in the peri-implant bone, by using the finite element analysis.

## Material and methods

### Model

**Fig. 1:**
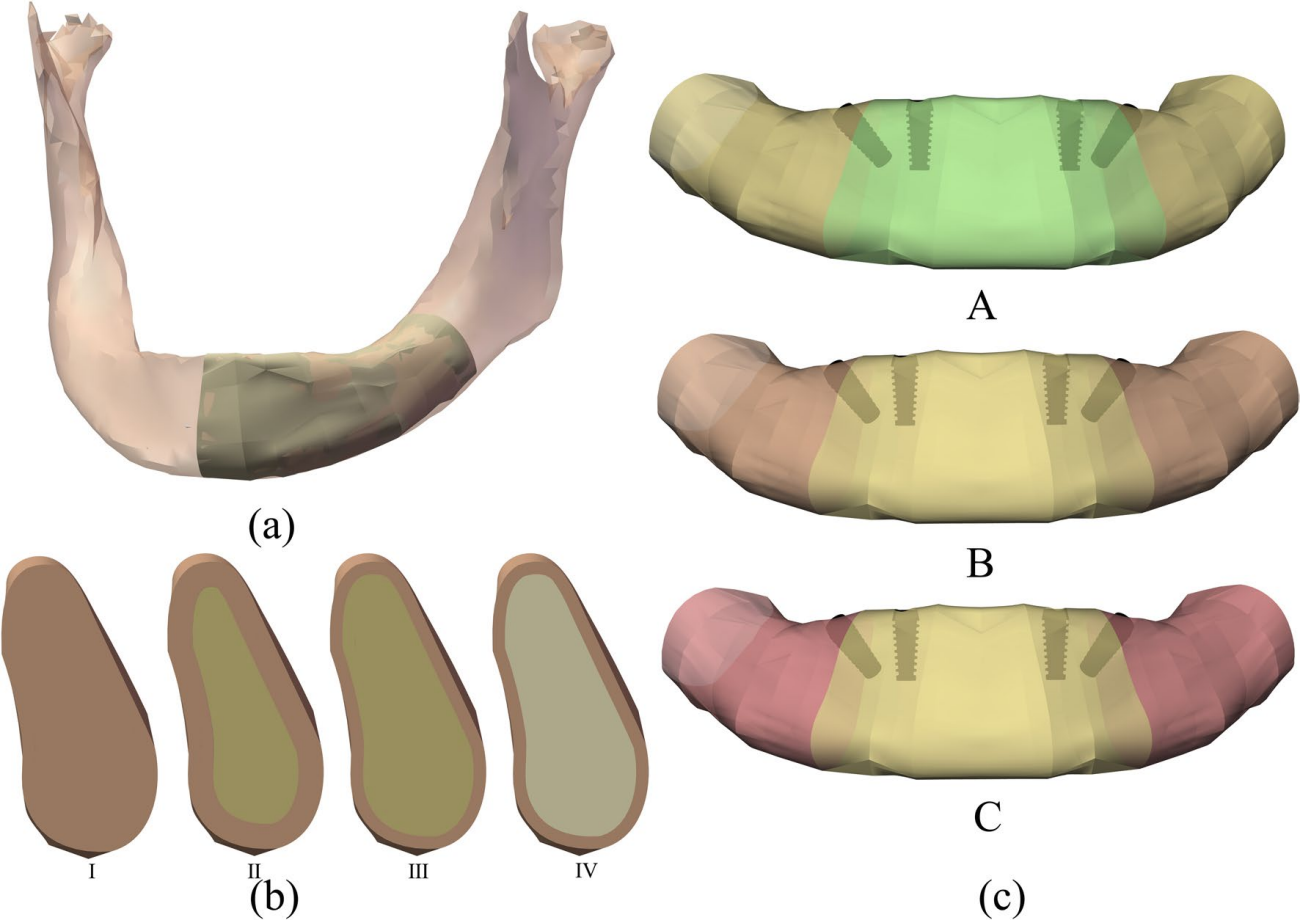
**a** The mandible model was created from a patient ‘s CBCT data, mirrored the left of the edentulous jaw. **b** Four types of bone were set as: Type I entire part assumed to be cortical; Type II 2mm thickness of cortical bone with inner high-density cancellous bone; Type III 1mm thickness of cortical bone with inner high-density cancellous bone; Type IV 1mm thickness of cortical bone with low-density cancellous bone. **c** Three kinds of jaws with different bone quality were: A anterior Type I bone and posterior Type II bone ; B anterior Type II bone and posterior Type III bone; C  anterior Type II bone and posterior Type IV bone.

A three-dimensional model of an edentulous atrophic mandible was created from a 62-year-old female patient’s CBCT data in Mimics Medical 21 software (Materialise NV, Leuven, Belgium). The mandible model was ported in the SolidWorks 2018 (Dassault Systèmes SolidWorks Corporation, Waltham, MA, USA), and mirrored the left part to create a symmetrical mandible model(Fig. 1a). For the need to create bone segments with different bone densities, an ideal model was created with a homogeneous layer of cortical bone in each region. Based on the classification proposed by Lekholm and Zarb [22] and Demenko’s study [23], cortical bone and cancellous bone were established separately in different sites (Fig. 1b). Three jaws with different bone densities in the anterior and posterior area were created (Fig. 1c).

**Fig. 2:**
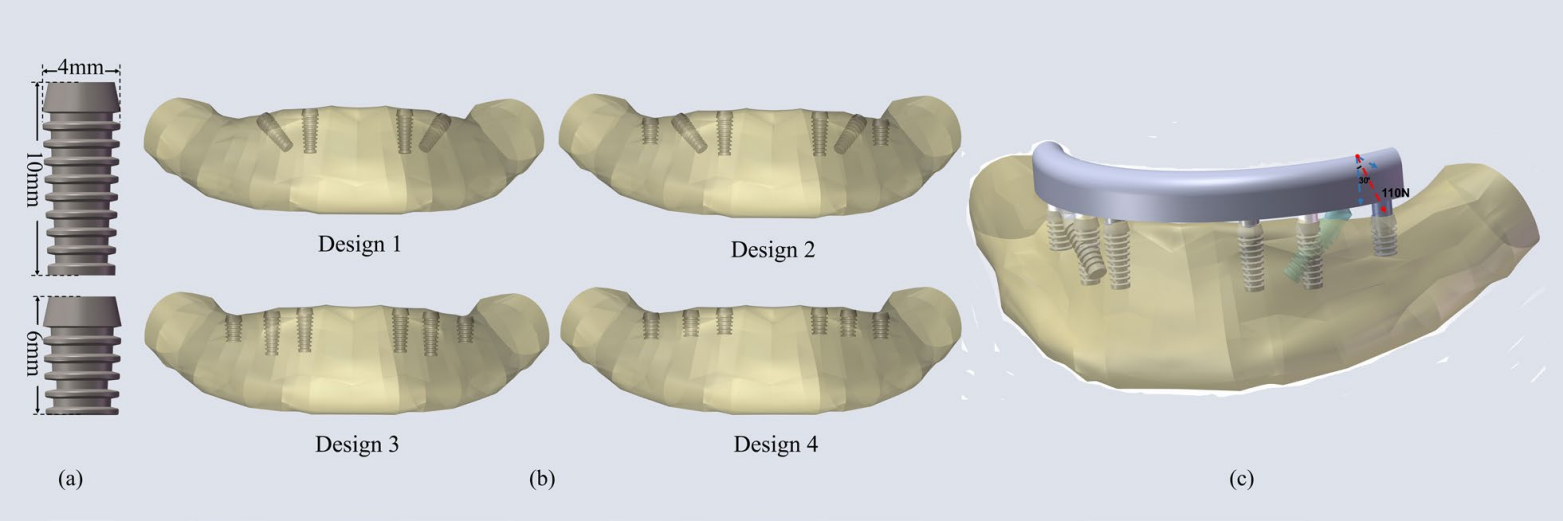
**a** two specifications of implants, **b** four implants restoration designs, **c** 110N oblique load applied on the framework at the left second premolar site

Drawing on the Bicon dental implants(Bicon, Boston, MA, USA), we built two specifications of implants, 4 × 6 mm short implant and 4 × 10 mm standard implant sharing the same thread depth and pitch, in SolidWorks (Fig. 2a).

There were four edentulous mandible implants restoration designs as follow (Fig. 2b):

1. 4 standard implants placed in interforaminal regions as the “all -on -4” concept, two straight implants at the lateral incisor sites and two tilted implants angled at 45 degrees lateral bilaterally inserted into the second premolars
2. 6 implants, straightly adding 2 short implants distally at the first molar sites to the 1) protocol
3. 6 implants, 4 standard implants straightly placed at lateral incisor and first premolar sites and two short implants at the first molar sites
4. 6 short implants placed at the lateral incisor, first premolar and first molar sites

The same simplified restoration and abutments models are created for the four designs, and all structures are assembled in Solidworks.

### Analysis

The assembled models were ported into the FEA software Abaqus 2018 (Dassault Systèmes SIMULIA Corp, RI, USA). All structures were considered linearly elastic, homogenous, and isotropic, except the mandible was set as orthotropy [13]. The material properties of all the models are listed in Table 1 [12, 24, 25].

**Table 1:** Material properties of the models

Materials	Young modulus (MPa)			Shearing modulus (MPa)			Poisson's ratio		
	E_x_	E_y_	E_z_	G_xy_	G_yz_	G_xz_	ν_xy_	ν_yz_	ν_xz_
Orthotropic									
Cortical bone	12600	12600	19400	4850	5700	5700	0.3	0.253	0.253
Cancellous bone (III)	1148	210	1148	68	68	434	0.055	0.01	0.322
Cancellous bone(IV)	692	145	692	68	68	261	0.048	0.01	0.322
Isotropic									
Titanium (implants, abutments)	110000			–			0.33		
Co-Cr alloy (framework)	218000			–			0.33		

A static 30-degree oblique load of 110 N in the buccal-lingual direction was applied at the second premolar site on the framework [26] (Fig. 2c). All the surface contacts were set bonded, which meant the bone-implant interface was considered complete osseointegration. The mandible was given a fixed boundary condition at the mentum and the attachments of the masseter and medial pterygoid. Quadratic tetrahedral elements (c3d10) were used to ensure the accuracy of the results, and the value of mesh size of the peri-implant bone was set up to 0.05 mm.

The stress–strain analysis was performed using maximum principal stress and strain. The peak values of maximum principal stress of peri-implant bone tissue were recorded. The volume of elements with strains over 3000 microstrans (με) in bone was counted for the risk of overload, according to the Frost's mechanostat theory [27]. The difference in stress and strain distribution was directly shown in nephograms.

## Results

### Maximum principal stress

**Fig. 3:**
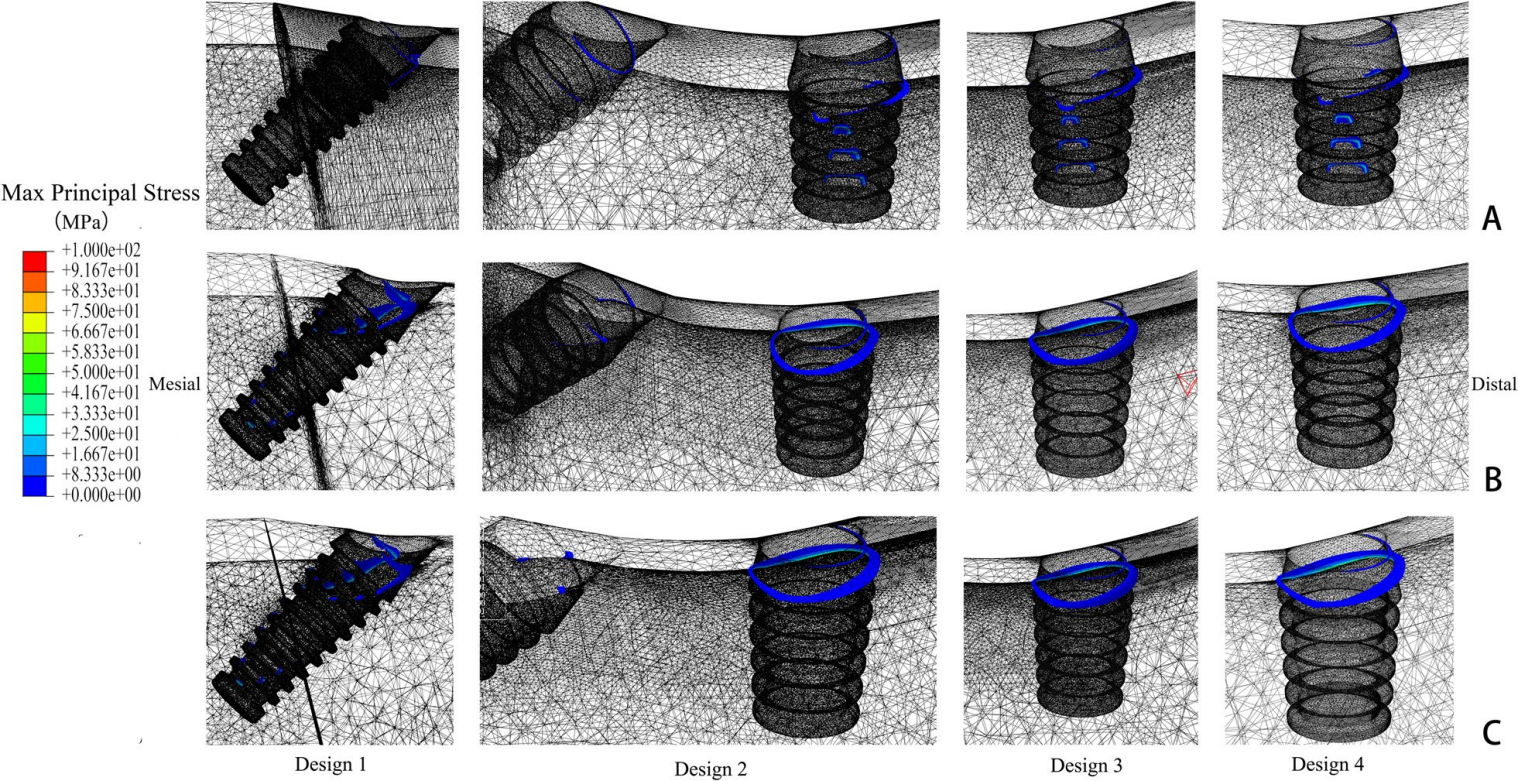
Distributions of maximum principal stress in bone around distal implants of loading side in different designs **A** high quality bone, **B** middle quality bone, **C** low quality bone

**Fig. 4:**
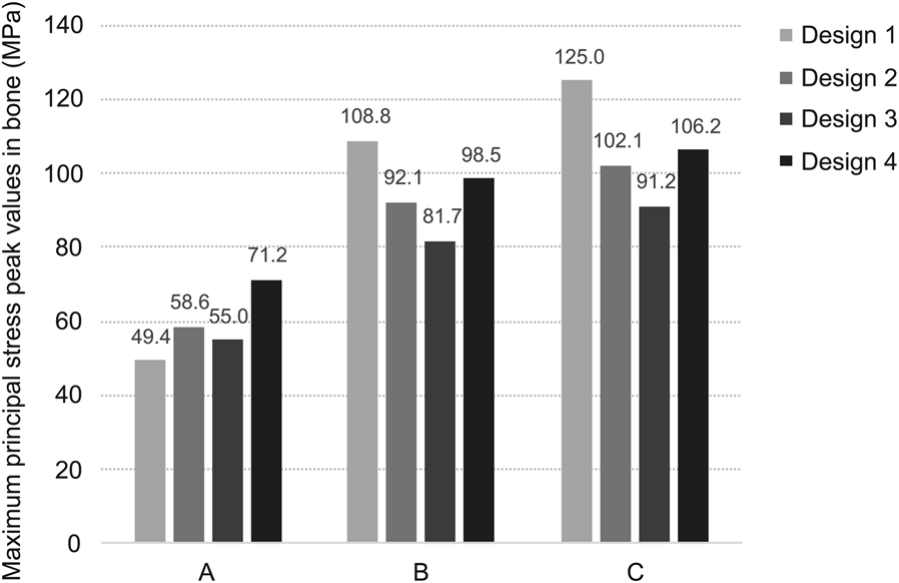
Peak values of maximum principal stress in peri-implant bone of four designs in different bone qualities

In four designs, the peak values of maximum principal stress were all in the cortical bone around the distal implants (Fig. 3). When the mandible had a high bone density, stress values were at a low level in four models. Stress mainly concentrated at the cortical and cancellous bone junction, like the neck of implants and threads deep into cortical bone. The highest peak stress value appeared in Design 4, followed by Design 2. While the “All-on-4” design had the lowest stress value (Fig. 4).

In the mandible with middle density, the peak stress value significantly increased in all groups. Design 1 had the highest value, followed by Design 4 and Design 2. Due to the reduction of cortical bone thickness, the middle of short implants in the posterior jaw was entirely surrounded by cancellous bone. Thus, stress only concentrated at the bone around the neck of short implants, and we can see the increase in the area of high-stress value.

As the posterior region was reduced to Class IV, the peak stress values increased further for each group. Design 3 had a lower stress value, but it still reached 91.22 MPa. Design 2 and Group 4 gave similar results, while Group 1 still shown the highest value.

Design 3 showed lower peak stress values in mandibles with different bone densities, yet it was similar to the other two groups with short implants except in the middle density. The results for Design 1 appeared to be more affected by the change of bone density. It should be noted that the peak stress values could only represent the condition of stress concentration points but not the overall situation. In the stress nephograms, the high stress areas just expanded slightly as the bone density decreased.

### Overload bone volumes

**Fig. 5:**
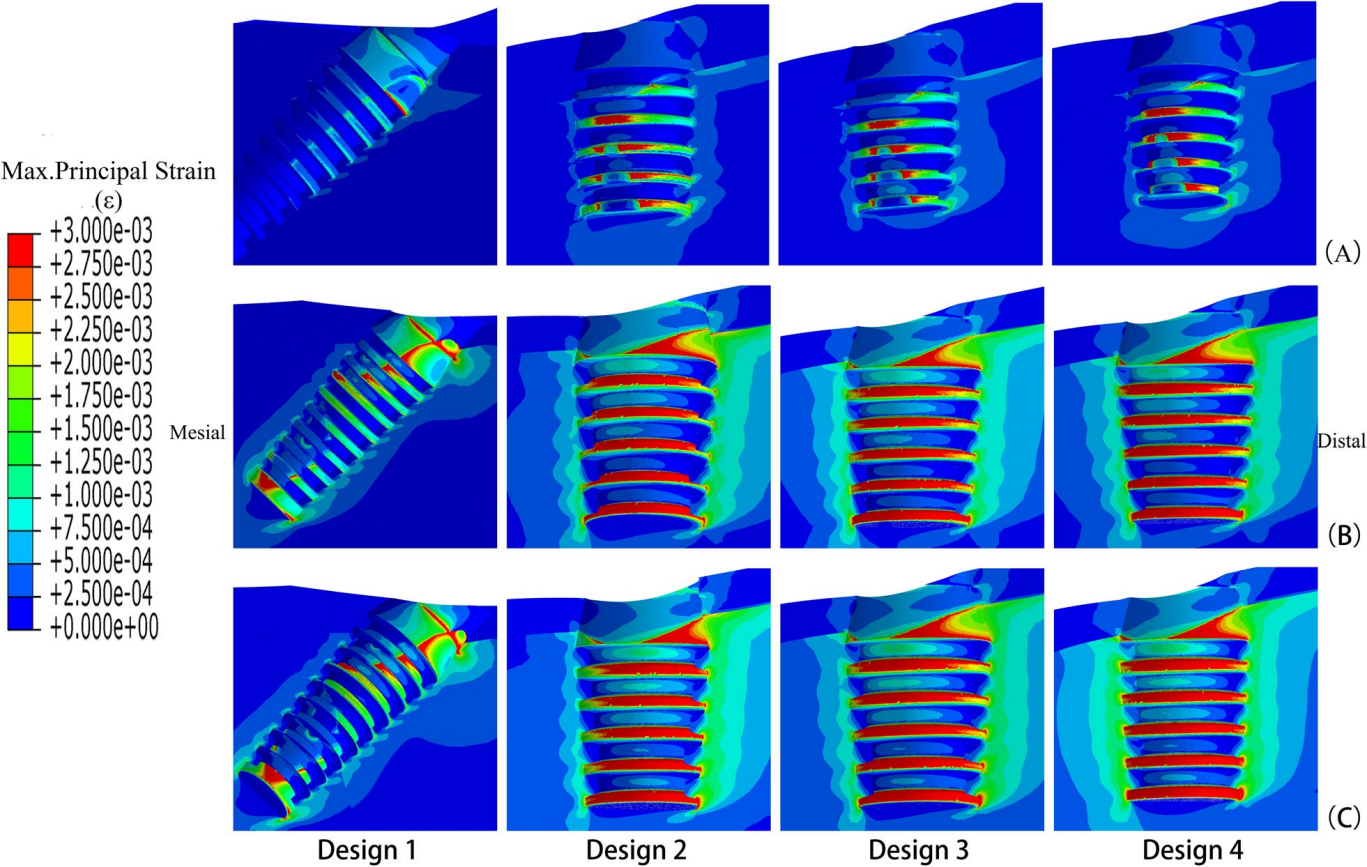
Maximum principal strains in bone around load-side distal implant of four designs **A** high quality bone, **B** middle quality bone, **C** low quality bone (overload area ≥ 3000 με)

**Fig. 6:**
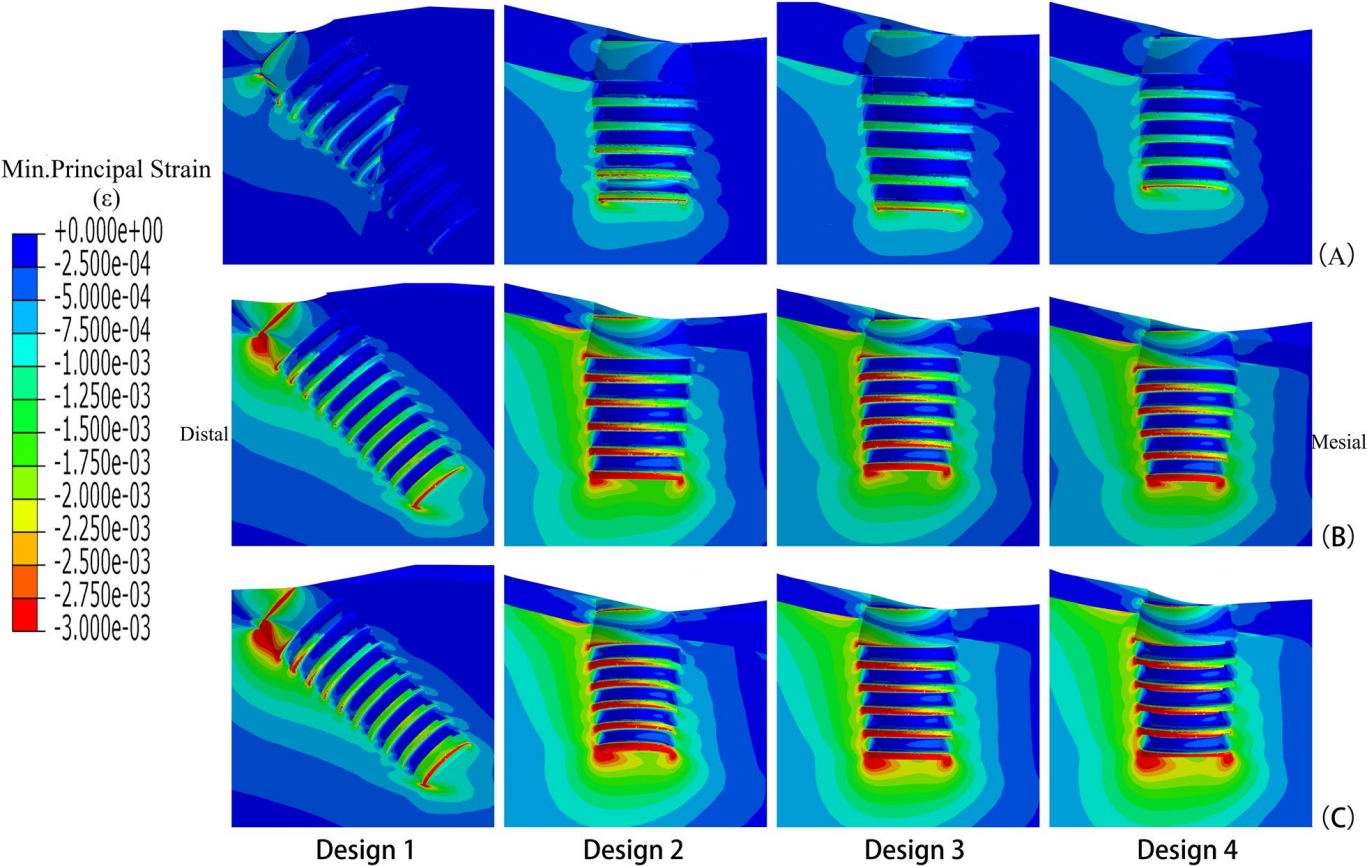
Minimum principal strains in bone around load-side distal implant of four designs **A** high quality bone, **B** middle quality bone, **C** low quality bone (overload area ≤ − 3000 με)

**Fig. 7:**
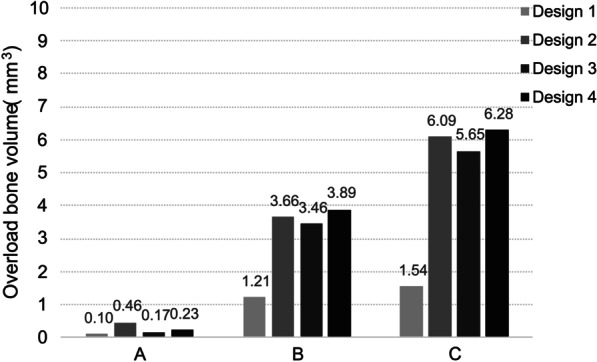
Overload bone volume of four designs in different bone qualities

Strain nephograms were plotted separately for under maximum principal stress (tensile stress) (Fig. 5) and minimum principal Stress (compressive stress) (Fig. 6). Unlike in the stress nephograms, the high strain area mainly concentrated at the cancellous bone around implant threads. Elements with strain over 3000με were calculated for it may disrupt the balance of bone remodeling and lead to bone resorption. The results of four designs in different density jaws were shown in the Fig. 7.

Within the high bone density jaws, there was little volume of bone with resorption risk in all four designs. Highest volume appeared in Design 2 as 0.46mm^3^. While in the two mandibles with lower density, Design 2 and Design 3 had the similar results. The volume of bone in Design 4 showed a higher increase with the change of bone density, in which the bone surrounding the distal short implant was under more risk of resorption than in others. Design 1 maintained the lowest level of overload bone volume.

## Discussion

The fretting of the implant caused by excessive immediate loading in the early stage can lead to the failure of the combination of implant and bone [28]. At the same time, excessive force is thought to cause resorption of the supporting bone even after the osseointegration formed. Previous studies have shown that, similar to periodontitis, excessive force will aggravate bone resorption under the effect of bacteria. However, there is not enough evidence to prove that excessive force can independently cause peri-implant bone resorption. Due to the differences of experimental animals, insertion sites and loading conditions (site, direction, size and frequency), the response of bone to loading can be density increased, accelerated resorption and no significant change. The reason may be that the force applied is not ‘excessive’ enough [29, 30].

In Frost’s mechanostat theory, load-bearing bones, including the mandible, can maintain the balance of density within a range of stress. Excessive force above the threshold can result in a decrease in bone density followed by bone resorption [27]. Frost used strain ranges as thresholds for each stage of bone remodeling, which are difficult to directly measure in vivo. FEA can provide information on the stress in each part of models, and is therefore well suited for the evaluation of bone tissue using this theory [25, 31].

In this study, the maximum principal stress peaks were distributed in the bone tissue around the distal implants on the loading side, whether there were four or six implants. Due to the significant difference of elastic modulus between cortical bone and cancellous bone, with a value ratio close to 10:1, the shielding effect made stress mainly concentrated in the cortical bone [10]. In the case of high bone density, the use of short implants in posterior area did not reduce tensile stress. As the bone quality decreased, the situation changed. The “All-On-4” group showed higher peak stress values than others, and Design 3 had the lowest stress value. This is consistent with the results of previous studies, since they had a similar bone quality setting [10, 11].

Comparing the volume of overload bone in four groups, the result was contrary to the stress value. As seen in the strain nephograms, the overload bone tissue is also mainly located around the distal implants. Owing to the horizontal component, the overload bone under tension and pressure was respectively on the lingual and buccal side of the jaw. More cancellous bone showed a tendency to resorption, related to its low stiffness. It underwent greater deformation even when it bore lower stress than cortical bone. The drop in bone density was accompanied by the decrease of the mechanical properties of the jaw, which lead to a significant increase of overload bone. In middle and low bone quality jaws, the volumes of overload bone in the latter three groups were times more than the “All-on-4” group, probably caused by the interaction between the smaller osseointegration area of the short implants and the bone density [19, 21]. Moreover, most of the four standard implants in Design 1 were placed in anterior regions with higher bone quality. In the presence of poor bone conditions, the implant length plays a more critical role in the distribution of stress than the implant diameter, which explains the greater range of overload strain in the jaw completely restored by short implants.

The “All-on-4” concept was invited to provide a simple, economicial and immediate loading scheme for the edentulous jaw. Though tilted implants were thought to put more stress on the supporting bone, they show a high success rate in years of follow-up [32]. In a priori studies, tilted placed distal implants in interforaminal regions helped reduced stress in alveolar bone, in cases that there was no sufficient bone in the posterior areas [33]. Takahashi et al.’s study [4] partially explicated the result associated with the cantilever length. When the load was at the end of the cantilever, the increase of the inclination angle of the end implant helped reduce the length of the cantilever and the moment of force, and the stress in peri-implant bone increased. Besides, when the load was kept at a certain distance from the end implant, the stress rose with the angle of the end implant increasing. The addition of short implants to the posterior area can shorten or even eliminate the cantilever, explaining the reduced stress in bone with the use of short implants in this study.

Short implants enable implant restoration in locations with limited vertical bone, avoiding the additional need for bone augmentation and the following complications [34–37]. Current literature shows that short implants have a high success rate, as well as similar marginal bone resorption and survival rates compared to longer implants with bone augmentation [38–40]. In the study results, short implants in the posterior area had similar bone stress and low risk of overloaded bone as "All-On-4" in the high bone quality mandible. In practice, the bone quality of the mandible is usually acceptable [41], and the use of short implants at the posterior is an alternative. Six or more implants give more options for restoration, such as segmented prostheses. The addition of posterior implants also helps maintain the restoration function in the event of implant loss, especially the distal-end implant [42].

In the study models with poorer bone quality, posterior areas showed a higher trend of bone resorption around short implants. The quality of maxillae, especially the posterior, is often unsatisfactory [41], which gives a warning for the use of short implants. Also, some clinical studies showed that short implants had a higher failure rate in the maxilla than in the mandible [43, 44]. Making the best use of the anterior area seems to be a better choice. While, the design should be selected case by case since the bone quality of the jaws varies considerably between individuals. Some meta-analysis studies show that there seems to be more uncertainty in the survival rate of short implants with the increase of time in function [43, 45, 46], yet these studies included insufficient randomized controlled evaluations and should be interpreted with caution.

Design 3 had better bone stress results among the three short implant designs. When the anterior bone is insufficient for vertical implants, tilted placed implants or short implants between the mental foramina are suitable without bone augmentation. Even in severely atrophic jaws, the only available design is to be supported entirely by short implants, and the bone height remaining also limits the specifications of the short implants [47]. Nonetheless, the bone stress behavior of the interforaminal designs is affected by various factors such as the bicortical anchorage, implant size, and implant distribution [48, 49]. Further experiments and clinical results are still required for validation.

The present study also has some limitations. As a method based on mathematical calculation, the results of FEA are influenced by various parameters, including but not limited to the model geometry, material properties, boundary conditions, loads, and model interface interactions [13]. Though the osseointegration interface followed the bonded settings as similar experiments, complete osseointegration was not in line with the actual situation. The setting of the static load saved the cost of calculation time, but it differed from the real dynamic chewing. Therefore, the results of this study are not precise values and can only be used as a basis for comparison within the designs. By the setting limitation in the study, the effects of different specifications and numbers of implants on stress need to be supplemented in the follow-up research.

In the four designs, the force didn’t lead to direct bone damage even in the lowest bone density [50]. The use of short implants in the posterior region can reduce the bone stress in edentulous restorations to a certain extent. However, in the posterior regions with low bone density, the bone around the short implants over the strain threshold increased and showed a tendency for resorption. In the case of sufficient bone, it is not advisable to completely replace standard implants with short implants. In situations where short implants are required in the posterior regions, it may be helpful to increase the number of short implants [51]. To be clear, the results of this study are only biomechanical results, and the outcome of short implants in edentulous jaws still needs to be proved by long-term clinical research.

## Conclusion

According to the result of the FEA study, posteriorly placed short implants can help the stress dispersion in the edentulous mandible with different bone qualities. Short implants can be an alternative in atrophic jaws with high bone quality. However, it brings more risks of bone resorption to use short implants in low bone quality regions. Therefore, the use of short implants in posterior jaws should be cautious.

## Data Availability

The data used and analysed during the current study are available from the corresponding author on reasonable request.
